# Clinical characteristics, HIV status, and molecular biomarkers in squamous cell carcinoma of the conjunctiva in Ghana

**DOI:** 10.1002/hsr2.108

**Published:** 2019-01-10

**Authors:** Lauren E. Merz, Osei Afriyie, Evelyn Jiagge, Ernest Adjei, Susan K. Foltin, Megan L. Ludwig, Jonathan B. McHugh, J. Chad Brenner, Sofia D. Merajver

**Affiliations:** ^1^ University of Michigan Medical School Ann Arbor Michigan USA; ^2^ Komfo‐Anokye Teaching Hospital Kumasi Ghana; ^3^ Department of Otolaryngology—Head and Neck Surgery Michigan Medicine Ann Arbor Michigan USA; ^4^ Program in Cellular and Molecular Biology University of Michigan Ann Arbor Michigan USA; ^5^ Department of Pathology Michigan Medicine Ann Arbor Michigan USA; ^6^ Department of Internal Medicine, Rogel Cancer Center Michigan Medicine Ann Arbor Michigan USA; ^7^ Department of Epidemiology, School of Public Health University of Michigan Ann Arbor Michigan USA

**Keywords:** CSCC, Ghana, HIV/AIDS, viral oncology

## Abstract

**Background and aims:**

Conjunctival squamous cell carcinoma (CSCC) varies in incidence geographically from 0 to 1 case per 100 000 per year globally. Additionally, the incidence of CSCC is known to increase 49% for every 10° decrease in latitude. Since the onset of the AIDS epidemic, there has been a trend of increasing incidence of CSCC in Africa, and despite relatively stable levels of ultraviolet (UV) exposure, there is an observed 12 times greater risk of developing CSCC when individuals are infected with HIV. In this study, we aim to analyze the clinical characteristics and biomarkers of CSCC in Ghana.

**Methods:**

In this study, a registry review of patients from January 2011 to May 2016 with CSCC at Komfo‐Anokye Teaching Hospital in Kumasi, Ghana, was performed (n = 64). Tumor blocks of the CSCC were analyzed for the expression of various biomarkers.

**Results:**

In this study, the median age of onset of CSCC is 46.5 years old (range of 20–90 y old). Fifty one and a half percent (n = 33) of the cohort is female. There is a low rate of smoking and alcohol use in our CSCC cohort. Thirty‐nine percent (n = 12) of Ghanaian men with CSCC are HIV−, while only 12% (n = 4) of women are HIV−. Fifteen patients had metastasis to lymph nodes or other tissues, and we observed a statistically significant relationship between HIV infection and metastasis (*P* = 0.027, chi‐squared test). We observed no statistically significant relationship between known prognostic CSCC biomarkers and HIV status, age, or tumor stage.

**Conclusion:**

Better characterization of CSCC could have a profound impact on the prevention, early identification, and treatment of CSCC in Africa. A retrospective chart analysis and collection of tumor samples can be challenging in this region due to methods of record keeping and stigma attached to clinical data such as HIV testing and smoking and alcohol use. As a result, in this study, data were often incomplete leading to inconclusive results and analysis that should be interpreted with caution. Future studies should consider a prospective study design that gathers clinical data in a standardized format and ensures fresh tissue from CSCC tumors.

## 1 INTRODUCTION

Conjunctival squamous cell carcinoma (CSCC), a subset of ocular surface squamous carcinoma, is a cancer of the eye with an incidence rate of 0 to 1 case per 100 000 per year globally.^1^ In the United States, this cancer typically effects elderly men (median age of 77) with high levels of ultraviolet (UV) exposure.^2^, ^3^ In fact, Newton et al showed that the incidence of CSCC increased by 49% for every 10° decrease in latitude, supporting the role of UV exposure as a major risk factor.^4^ Consistent with this finding, there are higher incidence rates of CSCC in equatorial areas of sub‐Saharan Africa than in the rest of the world (0‐5.3 cases per 100 000 vs 0‐1 case per 100 000, respectively).^5^ More recently, studies have also noted an apparent trend of increasing incidence of CSCC in sub‐Saharan Africa. For example, in Uganda, there were four cases of CSCC reported between 1960 and 1971 and 66 cases^6^, ^7^ reported between 1994 and 1997. Even accounting for better access to health care and increasing reporting of cancer in hospital‐based registries, these differences appear disproportionately larger than increased reporting alone would predict. In light of relatively small increase in levels of UV radiation exposure in the region, such increase in incidence has been putatively associated with the onset of the AIDS epidemic.^5^, ^8^ Consistent with the parallel increases in the incidence of both HIV and CSCC, a study also observed twelvefold greater risk of developing CSCC in patients infected with HIV.^9^

Although relationships have been drawn between CSCC and HIV/AIDS in countries in East Africa like Kenya and Uganda,^6^, ^7^, ^10^, ^11^, ^12^, ^13^ very few studies on CSCC in West Africa have been published. Additionally, while a relationship between CSCC and HIV/AIDS has been established in sub‐Saharan Africa, the pathogenesis and natural history of CSCC cases in HIV+ patients in Western Africa have not yet been described, limiting our understanding of causative risk factors for the disease. One potential causative factor is infection with human papillomavirus (HPV), although the literature has shown disparate findings regarding the role of HPV in the pathogenesis of CSCC in HIV+ patients. In one set of studies done in both Africa and the United States, evidence of HPV infection was found in most CSCC tumor samples.^13^, ^14^, ^15^, ^16^, ^17^, ^18^, ^19^, ^20^ In contrast, another set of studies from Africa as well as from other regions of the world such as the United States, Germany, Brazil, and Mexico has found no evidence of HPV in CSCC.^10^, ^21^, ^22^, ^23^, ^24^ In addition, prognostic tumor biomarkers like cyclin D, p16, estimated glomerular filtration rate (EGFR), and p53 have been described in CSCC in the United States, but these are not well studied in CSCC in Africa.^25^, ^26^, ^27^

This study seeks to establish the clinical characteristics of HIV+ and HIV− CSCC in Ghana through a retrospective chart review. To elucidate the pathogenetics of CSCC in Ghana, we analyze several tumor biomarkers as well as markers of HPV in a subset of CSCC samples. The natural history and pathogenesis of HIV/AIDS‐associated CSCC in Western Africa are an important question, especially as long‐term survivors of HIV infection are increasingly affected by cancer.^28^, ^29^ Better characterization of this cancer will have a profound impact on the prevention, early identification, and treatment of CSCC in Africa.

## 2 METHODS

### 2.1 Acquisition of clinical data

Approval was obtained from the Kwame Nkrumah University of Science and Technology/Komfo‐Anokye Teaching Hospital (KNUST/KATH) Ethics and Research Committee, and the Institutional Review Board (IRB) approved a waiver of consent. Charts were obtained of all patients who presented to the oncology or ophthalmology center with squamous cell carcinoma of the conjunctiva from January 2011 to May 2016. All other types of eye cancers were excluded. The data extracted included date of birth, sex, HIV status, comorbidities, smoking and alcohol behavior, date of diagnosis, history of present illness, tumor stage, tumor grade, descriptive factors of the tumor, therapy type, result of therapy, and survival. The data were cleaned, coded, and curated to eliminate duplication and inaccuracies or inconsistencies. It was then analyzed with Microsoft Excel 2011 as well as Statistical Package for the Social Sciences (SPSS) software, version 23.0 (SPSS, Inc., Chicago, IL).

### 2.2 Marker detection by immunohistochemistry

Fifty‐nine CSCC tumors in paraffin‐embedded block or slides from Ghanaian patients seen and/or treated at KATH were analyzed at the University of Michigan, under IRB approval. Twenty seven out of fifty nine (45.8%) of the samples have corresponding clinical information. For immunohistochemistry (IHC), formalin‐fixed paraffin–embedded (FFPE) blocks were submitted to the University of Michigan Histology Core for cutting and staining for p53, p16, cyclin D, and EGFR using an in‐house protocol.^30^ Reagents used include citrate buffer, pH 6.0 (Sigma, C9999), 3,3'‐diaminobenzidine (DAB) liquid chromagen solution B (Sigma, D6065), and DAB liquid buffer solution A (Sigma, D6190). Primary antibodies used were mouse p53, Ab‐6 (DO‐1) (Neomarker, MS‐187‐P), mouse p16, Ab‐1 (DCS‐50.1/47) (Neomarker, MS‐218‐P), mouse p16, Ab‐4 (16PO4) (Neomarker, MS‐887‐P1), rabbit cyclin D1 (SP4) (Neomarker, RM‐9104‐30), and EGFR (31G7) (Biotium, BNCH1194‐500). Rabbit Ab‐1 (1F8) (Neomarker MS‐107‐P1) and a mouse Ab (Thermofischer, catalog #31430) were used as secondary antibodies. Four independent replicates of immunohistochemical staining with parallel positive and negative control tissue were performed per sample. Slides were scored as positive or negative by two pathologists (O.A., J.M.) using the American Joint Committee on Cancer (AJCC) system.^31^ Biomarker positivity was defined as slides with greater than 75% of tumor cells with nuclear and cytoplasmic staining using the H‐score.^32^

### 2.3 Marker detection by DNA and RNA isolation

DNA isolation from FFPE samples was done as previously described^33^ using the two commercially available kits, Qiagen's AllPrep DNA/RNA FFPE Kit (catalog number 80234, Qiagen, Germantown, MD 20874) and the MACHEREY‐NAGEL GmbH & Co KG's NucleoSpin DNA FFPE XS (catalog number 740980.50, MACHEREY‐NAGEL GmbH & Co KG, Bethlehem, PA 18020, USA). Protocol 5.2 was followed using the xylene and ethanol to remove paraffin wax from tissue. We were unable to obtain high‐quality RNA from these blocks. Tissue digestion was carried out at room temperature overnight followed by supplementation with an additional 10 uL of Proteinase K provided by Qiagen kit and followed by the manufacturer's recommended protocol. DNA samples were first analyzed using a Nanodrop ND‐1000 for spectrum and Qubit High‐Sensitivity dsDNA (Invitrogen, Cat Q32854) for final concentration.

To determine the presence of HPV and identify the specific viral subtypes in each sample, genomic DNA extracts were assessed in quadruplicate using the multiplex competitive polymerase chain reaction (PCR), followed by amplicon‐specific single–base extension as used in the Head and Neck Squamous Cell Carcinoma (HNSCC) Cancer Genome Atlas (TCGA) project.^25^ Briefly, amplification of the heterogeneous E6 region of 15 high‐risk HPV types (HPV 16, 18, 31, 33, 35, 39, 45, 51, 52, 56, 58, 59, 66, 68, and 73), three low‐risk HPV types (HPV 6, 11, and 90), and a human glyceraldehyde‐3‐phosphate dehydrogenase (GAPDH) gene, as a control, was performed, followed by a shrimp alkaline‐phosphatase quenching. Multiplex single‐base‐extension reactions of type‐specific E6 probes create a mass differential for each extension product, which was analyzed on a matrix‐assisted laser desorption/ionization time‐of‐flight (MALDI‐TOF) mass spectrometer, an analysis, which was performed at the University of Michigan DNA Sequencing Core.

### 2.4 Statistical analysis

The data were analyzed with SPSS software, version 23.0 (SPSS, Inc., Chicago, IL), and a chi‐squared analysis was used on categorical data sets. Logistic regression was employed to analyze the relationship of various factors with age, applying a significance level of *P* < 0.05. A Bonferroni correction was applied for multiple testing of the biomarkers against clinical characteristics (six attributes, significance level of *P* < 0.0083 required).

## 3 RESULTS

### 3.1 Patient characteristics and cancer risk factors

Sixty‐four patients were diagnosed with CSCC from January 2004 to May 2016, but 49/64 (76.6%) of these cases were diagnosed between January 2010 and May 2016. The demographic information is outlined in Table 1. The mean age of the male patients is 47.2 ± 3.04 years old, and the mean age of the female patients is 51.9 ± 51.9 years old. We observed a significant relationship between HIV status (positive, negative, and unknown) and sex (chi‐squared test, *P* = 0.035). Thirty five out of sixty‐four (54.7%) patients had information on alcohol use recorded, and 34/64 (53.1%) had smoking data available. In this subgroup, 6/8 (75.0%) of the patients who used alcohol and 8/11 (72.7%) of the patients who smoked were HIV+. We observed a relationship between smoking and HIV infection (chi‐squared test, *P* = 0.029) as well as between alcohol use and HIV infection (chi‐squared test, *P* = 0.041). However, almost half of the patients had no recorded data on smoking and alcohol use, resulting in a small subgroup of patients available for this analysis.

**Table 1:** Clinical characteristics of patients with conjunctival squamous cell carcinoma (CSCC)

Variables	HIV+ (N = 32)	HIV− (N = 16)	Unknown HIV Status (N = 16)	*P* value
Sex				0.035^1^
Male (N = 31)	14	12	5	
Female (N = 33)	18	4	11	
Age, y				0.241^2^
Mean	46.1	52.96	48.1	
Standard deviation	2.7	5.1	6.4	
Range	35‐58	25‐90	20‐75	
Smoking (N = 34)				0.029^1^
Current or former	8	1	2	
None	6	10	7	
Alcohol use (N = 35)				0.041^1^
Current or former	6	1	1	
None	7	11	9	

1 Chi‐squared test.

2 Logistic regression.

Chi‐squared analysis was used to compare sex and HIV status (*P* = 0.035), smoking behavior and HIV status (*P* = 0.029), and alcohol use and HIV status (*P* = 0.041). Mean age, standard deviation, and age range are also recorded by HIV status.

### 3.2 HIV status and history of HIV treatment

**Figure 1:**
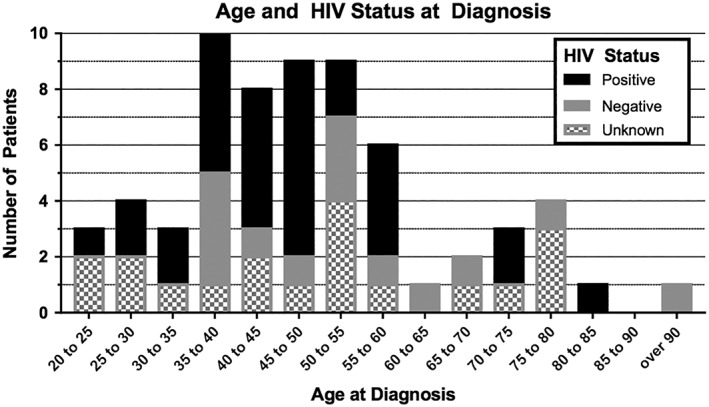
Age and HIV status at diagnosis. The ages are arranged in bins of 5 years, and the HIV status is indicated by the colors. We observed no statistically significant relationship between age at diagnosis and sex or between age at diagnosis and HIV status (logistic regression, *P* = 0.419; *P* = 0.241, respectively). However, we note that no patient below age 35 had a positive HIV test

Sixteen out of sixty four (25%) of the patients had no HIV testing recorded, 16/64 (25%) had negative HIV tests, and 32/64 (50%) had positive HIV tests. The mean age (±SD) of the HIV+ patients at CSCC diagnosis was 46.1 (±2.7) years old (median: 45 y old, range: 35‐58), and the HIV− patients had a mean age of 52.96 (±5.1) years old (median: 51 y old, range: 25‐90) at diagnosis. We observed no relationship between age at diagnosis and sex or between age at diagnosis and HIV status (logistic regression; *P* = 0.419; *P* = 0.241, respectively). The distribution of age at diagnosis with HIV status is depicted in Figure 1. Of note, 52/64 (81.2%) of the patients with CSCC are under 60 years old. Of the patients under 60 years old, only 10/52 (19.2%) have a negative HIV test.

### 3.3 Clinical characteristics and natural history of conjunctival cancers in Ghana

**Figure 2:**
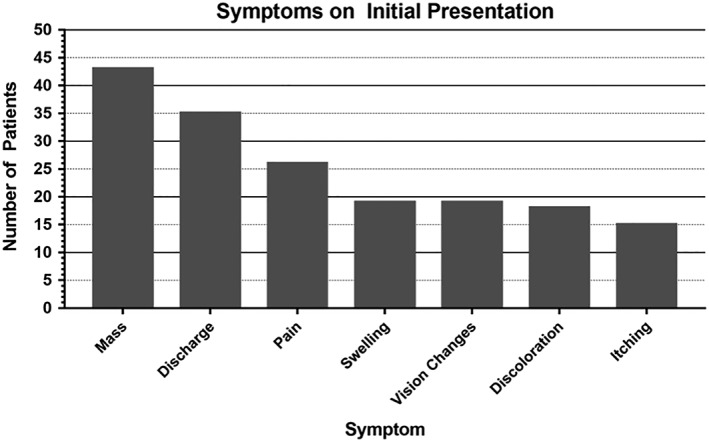
Symptoms on initial presentation. Patient symptoms on initial presentation with conjunctival squamous cell carcinoma (CSCC). The most common complaint was the presence of a mass (n = 43) followed by discharge (n = 35), pain (n = 26), swelling (n = 19), vision changes (n = 19), eye discoloration (n = 18), and itching (n = 15)

Patients first presented to the hospital 3 weeks to 5 years after the initial symptom was noted (median: 1 y; interquartile range [IQR]: 0.35‐1.5). After diagnosis, the most common symptoms patients complained of are shown in Figure 2. The different types of discharge were stratified: 17/35 (48.6%) complained of purulent drainage, sanguineous drainage, or a combination of the two; 11/35 (31.4%) complained of tearing, and 8/35 (22.9%) had unspecified types of discharge. Additionally, 16/64 (25%) patients complained of constitutional symptoms like weight loss, anorexia, or cough. Eleven out of sixteen (68.8%) of these patients were confirmed HIV positive (chi‐squared test, *P* = 0.011). At the last recorded patient visit in each individual chart, 47/64 (73.4%) of patients were generally well (defined as written note of “generally well,” condition indicated to be steadily improving, treatment effective/on schedule), and only 1/64 (1.6%) patient was known to be deceased.

### 3.4 Treatment modality and recurrences

Fifty five out of sixty four (85.9%) of the patients had treatment modality recorded. Thirty out of fifty‐five (54.5%) patients received single therapy (excision, radiotherapy, or incisional biopsy), 17/55 (30.5%) received adjuvant therapy (excision or incisional biopsy with radiotherapy or chemotherapy), and 8/55 (14.5%) had total removal of the eye (exenteration or enucleation). There were 22 recorded recurrences (34.4%), with median recurrence appearing 1 year after initial diagnosis (IQR: 0.42‐1.4). We found no relationship between recurrence and treatment modality in this cohort (chi‐squared test, *P* = 0.148). Of patients with recurrences, 10/22 (45.5%) were HIV+, and 4/22 (18.2%) were HIV−. We found no statistically significant relationship between HIV status and tumor recurrence (chi‐squared test, *P* = 0.856).

### 3.5 Tumor stage, grade, and metastasis

Forty seven out of sixty four (73.4%) of the patients had tumor grade indicated in their pathology report. Twenty out of forty seven (42.6%) of the tumors were well differentiated, 19/47 (40.4%) were moderately differentiated, and 8/47 (17.0%) were poorly differentiated. We found no statistically significant relationship between grade and HIV status (chi‐squared test, *P* = 0.734). Fifty seven out of sixty‐four (89.1%) patients had tumor stage indicated. We observed no statistically significant relationship between tumor stage and HIV status (chi‐squared test, *P* = 0.189). Additionally, 15 patients had metastasis to lymph nodes or other tissue such as brain or sinuses. The patients' demographic information is listed in Table 2. We observed a statistically significant relationship between HIV infection and metastasis to lymph nodes and/or other tissues (12 HIV+, 1 HIV−, two unknown HIV status) when compared with patients without metastasis (20 HIV+, 15 HIV−, 14 unknown HIV status) (chi‐squared test, P = 0.027)

**Table 2:** Metastasis to lymph nodes and other tissues and HIV status

Variables	HIV+ (n = 12)	HIV− (n = 1)	Unknown HIV Status (n = 2)	
Age			
Median	45.5	51	52.5
IQR	35.5‐50.25	‐	‐
Sex			
Male (N = 5)	4	1	0
Female (N = 10)	8	0	2
Metastasis location			
Lymph node	7	1	2
Brain	3	0	0
Sinuses (frontal/ethmoid)	2	0	0

Abbreviation: IQR, interquartile range. Fifteen out of sixty‐four (23.4%) patients had metastases to lymph nodes or other tissue such as brain or sinuses. The HIV status, sex, location of metastasis, and median age with IQR of the patients with recorded metastasis are listed in the table.

### 3.6 Molecular markers of CSCC

Twenty seven out of fifty nine (45.8%) of the CSCC tumor samples yielded enough viable tissue for analysis and had corresponding clinical data. Of these 27 patients who had samples that were analyzed for biomarkers, 12/27 (44.4%) were female with a mean age of 48.8 years old (median: 45 y old, range 20‐90), and 13/27 (48.1%) were HIV+, with 8/27 (29.2%) with unknown HIV status. Of note, there is a lower percentage of females in this subset than in the entire sample (44.4% vs 51.5%) and a greater percentage of unknown HIV status in this subset (29.2% vs 25%). Fifty‐nine CSCC samples were analyzed, but only 27 samples had corresponding clinical information. Importantly, all 59 CSCC samples were analyzed for multiple biomarkers, but not every sample had results for every biomarker. Additionally, the demographic data are different in this subset than in the general sample, as mentioned above.

**Figure 3:**
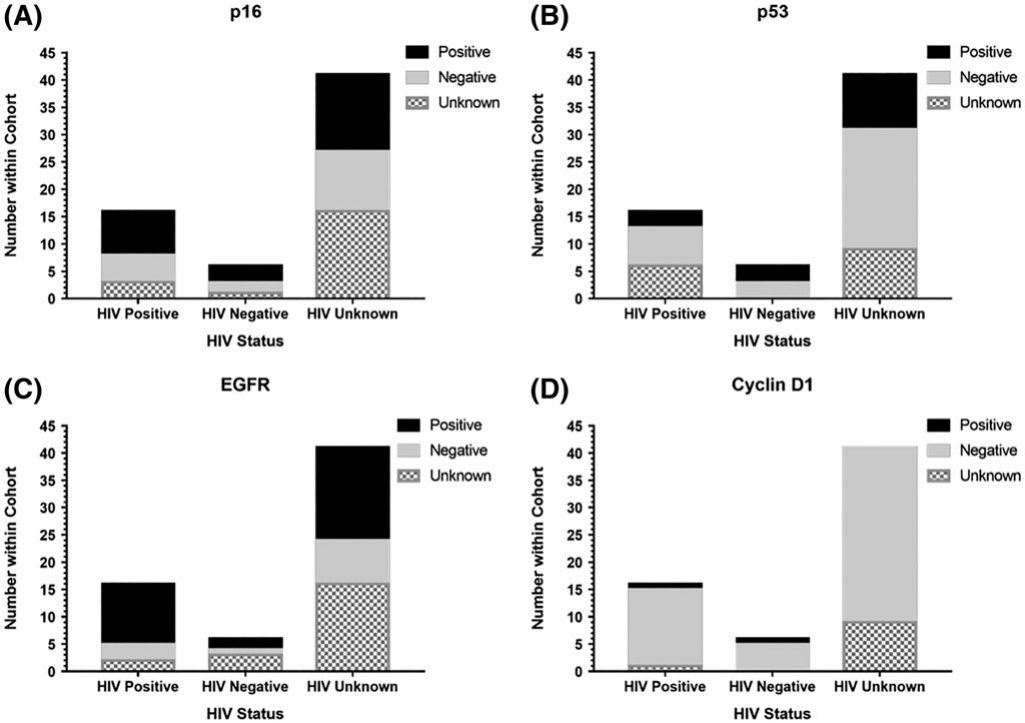
Presence of tumor biomarkers stratified by HIV status. The results of the tumor biomarker analysis are graphed and stratified by HIV status. All 59 CSCC samples were analyzed for p53, p16, EGFR, and cyclin D, but not every sample yielded a result for every biomarker. As such, the number of samples within each biomarker graph does not always add up to 59. We observed no significant relationship between any of the tumor biomarkers and HIV status, sex, or stage of the tumor. However, only 27 samples had corresponding clinical data

**Figure 4:**
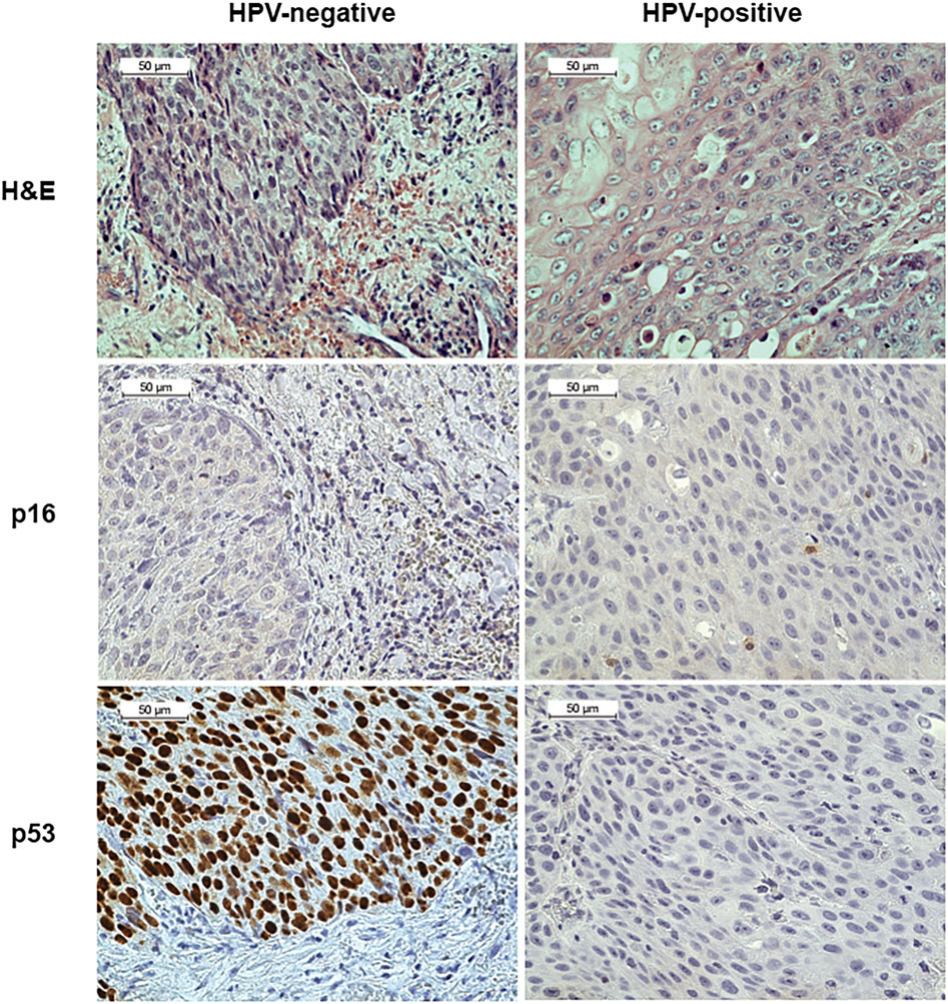
Immunostaining does not correlate with human papillomavirus (HPV) status in our conjunctival squamous cell‐carcinoma (CSCC) cohort. Four independent replicates of immunohistochemical staining with parallel positive and negative control tissue were performed per sample, and representative images from p16 and p53 staining from an HPV positive case and HPV negative case are shown. We expected to observe p53 expression in the HPV negative cases and p53 suppression in HPV positive cases as p53 is known to be suppressed in HPV infection. Indeed, p53 expression is seen in the HPV negative cases, and p53 suppression is seen in the HPV positive case. Additionally, we expected to see p16 expression in the HPV positive case as p16 is a frequent marker of HPV infection in cells. However, p16 is negative in both the HPV positive case and HPV negative case. It is possible that p16 expression is not an accurate marker for HPV infection in these samples

Molecular events that are commonly altered in HNSCCs of other subsites including p16, p53, EGFR, and cyclin D1 as well as HPV status were analyzed. Overexpression of cyclin D1 is known to cause p16 overexpression,^17^ but we found no relationship between p16 and cyclin D in this cohort (chi‐squared test, *P* = 0.881). In addition, EGFR is typically overexpressed in CSCC in the United States and Europe and is often induced through smoking.^27^ However, we found no relationship between EGFR and smoking in this cohort (chi‐squared test, *P* = 0.292). The relationship between individual biomarkers and clinical characteristics such as HIV status, smoking and alcohol use, sex, and age is shown in Table 3. The results of the biomarkers that produced valid results from PCR Mass Array (MA) are shown in Figure 3. It was postulated that patients with CSCC would show coinfection with HPV and HIV.^17^, ^18^, ^34^ However, 0/27 (0%) samples with clinical data were HPV positive. Two out of fifty nine (3.4%) of the total CSCC tumor samples are HPV16+. The p53 is typically suppressed in HPV+ tumors,^35^ and both HPV16+ tumors in our CSCC cohort show decreased p53 expression as expected. Conversely, p16 alone is often viewed as a marker for HPV infection in a cell,^36^ but there were 21/59 (35.6%) p16+ samples, and none of the p16+ samples were also HPV+. Furthermore, both HPV+ CSCC samples had negative p16 staining on IHC. Therefore, p16 immunostaining did not seem to correlate with HPV status in our CSCC cohort, as seen in Figure 4. However, these results once again must be interpreted with caution as only a small subset of patients had tumors available for testing and the samples that were successfully analyzed did not always yield results for every biomarker.

**Table 3:** Comparison of biomarkers and clinical characteristics

	Cyclin D	p16	p53	EGFR
HIV status	0.596	0.699	0.639	0.929
Sex	0.859	0.653	0.772	0.171
Grouped stage	0.009	0.252	0.677	0.687
Age	0.824	0.488	0.763	0.583
Smoking	0.612	0.707	0.849	0.131
Alcohol use	0.732	0.707	0.792	0.793

The *P* values of biomarkers compared with clinical characteristics are shown. A Bonferroni correction was applied for multiple testing (six attributes, *P* < 0.0083 required for significance). We found no statistically significant relationship between any of the tumor biomarkers and clinical characteristics.

Abbreviation: EGFR, Epidermal growth factor receptor. Chi‐squared analysis was used to compare individual biomarkers and HIV status, sex, grouped stage (stages 1 + 2 vs stages 3 + 4), smoking behavior, and alcohol use. Logistic regression was used to compare biomarkers to age.

## 4 DISCUSSION

This study was the first to assess the demographics, clinical characteristics, and tumor biomarkers in CSCC in Western Africa. Previous studies on CSCC in Africa describe that clinical characteristics of all ocular neoplasms do not separately analyze the characteristics of HIV+ and HIV− patients or are conducted in Eastern Africa.^35^, ^37^, ^38^, ^39^, ^40^, ^41^ Additionally, other studies of CSCC in Africa report a higher rate of HIV infection in patients with CSCC than in the general population, ranging from 50% to 76.9% of the CSCC population with HIV infection.^5^, ^37^, ^38^, ^39^, ^42^ This was supported by the findings in our study, as 32/64 (50%) of the patients with CSCC are HIV+ while the incidence of HIV infection^43^ in Ghana is 1.47%.

In the United States, CSCC is seen two to three times more in men than women, and it presents at a median age of 77 years old.^3^ However, in Ghana, over half (51.3%) of the patients diagnosed with CSCC are female, and the median age is 46.5 years old, with some patients presenting as young as 20 years old. This is also consistent with some studies of CSCC performed in Eastern Africa that report that over half of their CSCC cohorts are female, with a mean age at diagnosis^37^, ^38^, ^39^, ^40^, ^42^, ^44^ in the 30s to 50s. Additionally, in the general Ghanaian population, the proportion of people with HIV infection peaks between the ages of 35 to 45 years old, at^45^ approximately 3%. However, we found no significant relationship between age and HIV status in our cohort, but we are limited by small sample size and many patients with unknown HIV status, which could have resulted in selection bias. Of note, in our cohort of patients with CSCC, only 10/52 (19.2%) of patients below 60 years old had a negative HIV test, and no patients below age 35 had a negative HIV test. In addition, only 3/12 (25%) of patients above 60 years old had positive HIV tests. The difference in the age and sex of patients with CSCC in the United States and Ghana may be driven by HIV infection rates in Ghana; as there appears to be a significant relationship (chi‐squared test, *P* = 0.035) between female sex and HIV infection, only 19.2% of patients below 60 years old had a negative HIV test, and no patient below 35 years old has a negative HIV test.

The CSCC cohort reported 3.1% current smokers and 7.8% former smokers, which closely mirrors the general Ghanaian population data,^46^ suggesting that smoking is not a major risk factor in the pathogenesis of CSCC in Ghana. In the United States, smoking and alcohol use are important risk factors for HNSCC,^47^, ^48^ but there does not seem to be an increased uptake among patients with CSCC in Ghana. We observed a significant relationship between smoking and HIV infection (chi‐squared test, *P* = 0.029) as well as between alcohol use and HIV infection (chi‐squared test, *P* = 0.041) in this cohort. However, the patient charts at KATH seldom reported detailed alcohol exposures making it difficult to differentiate alcohol use from abuse. Additionally, this analysis is limited by the fact that only 35/64 (54.7%) of patients had information on alcohol use recorded and only 34/64 (53.1%) had smoking data available, leaving the data set open to selection bias. Analysis with more complete patient data is required to draw further conclusions.

The clinical presentation of CSCC in HIV+ and HIV− patients in Ghana was also examined in this study. Most patients had symptoms for 1 year before presentation, and the most common symptom at presentation was a mass. After diagnosis, the most common symptoms included discharge, eye pain, and headache. This presentation is consistent with CSCC in the United States as well as in Africa.^26^, ^38^, ^39^, ^42^, ^44^ Additionally, 16/64 (25%) of the Ghanaian cohort had at least one constitutional symptom, and we observed a significant relationship between constitutional symptoms and HIV infection (chi‐squared test, *P* = 0.011). Conversely, constitutional symptoms are rare in patients with CSCC in the United States and have not previously been reported in Africa.^26^ HIV infection could cause a more systemic and aggressive manifestation of CSCC. However, HIV+ patients are also more susceptible to infection, so the reported constitutional symptoms could be related to an underlying chronic infection rather than the CSCC.

Recurrence after initial treatment is common in both the United States and Ghana because of ambiguous tumor margins as well as desire to preserve the eye.^49^ Twenty two out of sixty‐four (34.4%) patients reported recurrences. Recurrences were diagnosed a median of 1 year after initial diagnosis, mirroring the United States.^3^ Nine out of eighteen (50.0%) patients with excision‐only therapy reported recurrences compared with 29% recurrence rate in the United Stated with monotherapy.^3^ We found no significant relationship between treatment modality and recurrence or between HIV status and recurrence. However, follow‐up visits were scarce, so there may be recurrences that went unreported.

This study found no significant relationship between HIV status and tumor stage (chi‐squared test, *P* = 0.189). However, 15/64 (23.4%) patients showed metastasis to regional lymph nodes and/or other sites like brain or sinuses, and there was a significant relationship between HIV infection and metastasis (chi‐squared test, *P* = 0.027). However, only 47/64 (73.4%) had information on stage or metastasis recorded. This led to a subgroup analysis of the cohort, which could have resulted in selection bias. In the United States, CSCC rarely (<1%) metastasizes to regional lymph nodes or distant sites.^2^ Although these conclusions must be interpreted with caution, it is possible that HIV infection may allow a more aggressive form of CSCC that results in more metastasis and spread than HIV− CSCC.

Biomarkers including p16, p53, EGFR, and cyclin D1 as well as various types of HPV were analyzed from the tumor samples. Unfortunately, only 27/64 (42.2%) of the patients with CSCC had tumor samples available for analysis, which makes this aspect of the study susceptible to selection bias. In addition, there is a lower percentage of females in the subset with tumor samples available for analysis than in the entire cohort (44.4% vs 51.5%) as well as a greater percentage of unknown HIV status in this subset compared with the whole cohort (29.2% vs 25%) further raising concerns about selection bias. We found no significant relationship between any of the biomarkers and clinical characteristics such as age, HIV status, or alcohol use and smoking. It was originally hypothesized that HPV infection would be comorbid with HIV infection.^17^, ^18^, ^34^ However, only 2/59 (3.4%) of the CSCC samples tested positive for HPV, and neither of those HPV+ samples have corresponding clinical data. Of note, p16 overexpression is a common marker for HPV infection in a cell.^36^ There are far more p16+ than HPV+ samples in our cohort, and immunostaining for p16 does not correlate with HPV status in our CSCC samples. This suggests that more CSCC tumors in this cohort may be HPV+, but we are limited by our ability to capture enough DNA for valid testing, and the method for detecting HPV directly may not be sufficiently sensitive for such small DNA aliquots. Additionally, the small subset of patients with both tumors available for analysis and clinical data opens this study to significant selection bias. It is also possible that we are overlooking significant relationships because of the small sample size and even smaller subset of patients with tumors available for analysis. Overall, there are no conclusive biomarker data from this cohort of Ghanaian patients with CSCC secondary to incomplete clinical data and only a small subset of patients with tumors available for analysis.

As highlighted throughout the manuscript, this study had limitations. It was often difficult to obtain both clinical information and tumor sample for the same patient. For example, while 209 cases of CSCC were identified through an oncology database and the records of a KATH pathologist, only 64 charts of patients with CSCC were physically located (30.6%), and only 35 corresponding tumor samples were found (54.7%). Only 27 samples were viable due to mislabeling as CSCC, corruption with bacteria, or too brittle for testing (77.1%). Thus, only 27/209 (12.9%) of all identified CSCC cases at KATH between January 2011 and May 2016 had both sufficient clinical data and tumor sample available for biomarker analysis. This amount of missing data leaves the study susceptible to selection bias. Furthermore, the medical charts identified and located were often incomplete, resulting in unknown pieces of important data like HIV status. This led to inconsistencies in the quantity and quality of information on each patient and further leaves the data set susceptible to selection bias. Additionally, the small tumor size made it difficult to retrieve sufficient DNA to assess HPV infection, and the method used may not be sensitive enough to detect small markers of HPV infection. In this region, a retrospective chart analysis and collection of tumor samples can be challenging due to methods of record keeping and stigma attached to clinical data such as HIV testing and smoking and alcohol use. We were only able to obtain clinical information and viable tumor samples in 12.9% of all identified CSCC cases at KATH between 2011 and 2016. This significantly limited our analyses and conclusions. Future studies should consider a prospective study design that gathers clinical data in a standardized format and ensures fresh tissue from CSCC tumors for biomarker analysis.

## 5 CONCLUSION

In conclusion, this study highlighted the equal incidence of CSCC in males and females in Ghana as well as the 15.6‐fold higher HIV infection rate in the CSCC cohort compared with the general Ghanaian population. We also found a significant relationship between female sex and HIV infection in this cohort. Smoking, a known risk in upper aerodigestive‐tract HNSCC in the United States, has no greater incidence in CSCC cohort than in the general Ghanaian population, suggesting that smoking is not a major risk factor in CSCC in Ghana. However, only 53.1% of patients had smoking data recorded, leaving the data set susceptible to selection bias. Additionally, HIV+ CSCC patients are significantly more likely to have metastasis and a history of constitutional symptoms than the HIV− CSCC patients, suggesting that HIV+ CSCC may be a more aggressive cancer than HIV− CSCC. Furthermore, we postulated that there would be a relationship between HIV and HPV, p53, cyclin D, EGFR, or p16, but we found no relationship between these tumor biomarkers and sex, stage, or HIV status. However, these data should be interpreted with caution as patient information was often incomplete and only a small subset of patients had tumor samples available for analysis. This results in possible selection bias, and the small sample size raises the possibility that significant relationships may have been overlooked. More conclusive results could be obtained in a prospective study in which patients' HIV status is noted and fresh tissue from their tumor sample is collected for biomarker analysis.

## CONFLICTS OF INTEREST

No potential conflict of interest was reported by the authors. The supporting funds had no involvement in the study design, collection, analysis and interpretation of data, the writing of the report, or the decision to submit for publication.

## AUTHOR CONTRIBUTION

Conceptualization: Sofia Merajver, Chad Brenner, Jon McHugh, Lauren Merz

Data curation: Sue Foltin, Lauren Merz, Osei Afriyie

Formal analysis: Lauren Merz, Chad Brenner, Megan Ludwig

Investigation: Lauren Merz, Evelyn Jiagge, Ernest Adjei, Osei Afriyie

Methodology: Chad Brenner, Jon McHugh, Osei Afriyie

Resources: Jon McHugh, Chad Brenner

Supervision: Sofia Merajver, Chad Brenner

Validation: Sue Foltin, Chad Brenner

Visualization: Sue Foltin, Chad Brenner, Lauren Merz

Writing—original draft: Lauren Merz

Writing—review and editing: Lauren Merz, Megan Ludwig, Chad Brenner, Sofia Merajver, Jon McHugh

Dr J. Chad Brenner and Dr Sofia D. Merajver had full access to all of the data in this study and take complete responsibility for the integrity of the data and the accuracy of the data analysis.
